# The relationship of oleic acid/albumin molar ratio and clinical outcomes in leptospirosis

**DOI:** 10.1016/j.heliyon.2021.e06420

**Published:** 2021-03-08

**Authors:** Caroline Azevedo Martins, Maria Conceição B dos Santos, Cassiano Felippe Gonçalves-de-Albuquerque, Hugo Caire Castro-Faria-Neto, Mauro Velho Castro-Faria, Patricia Burth, Mauricio Younes-Ibrahim

**Affiliations:** a Laboratório Integrado de Nefrologia, Department of Internal Medicine, Medical Sciences School, State University of Rio de Janeiro, Brazil; b Laboratório de Imunofarmacologia, Oswaldo Cruz Institute, Fiocruz, Rio de Janeiro, Brazil; c Laboratório de Enzimologia e Sinalização Celular, Department of Cellular and Molecular Biology, Federal Fluminense University, Niteroi, Brazil; d Laboratório de Imunofarmacologia, Departamento de Bioquímica, Universidade Federal do Estado do Rio de Janeiro, Rio de Janeiro, Brazil; e Departamento de Medicina, Pontifícia Universidade Católica, Rio de Janeiro, Brazil

**Keywords:** Leptospirosis clinical outcome, Acute lipotoxicity, Lipidome, Oleic acid/albumin, Na/K-ATPase

## Abstract

Human leptospirosis is an acute infectious zoonosis presenting specific lipid disorders. Previous *in vitro* studies showed both leptospira glycolipoprotein endotoxin, and high oleic acid levels were associated with Na/K-ATPase inhibition that is amplified by the reduction of circulating albumin levels. In this study, we aimed to investigate the relationship of oleic acid/albumin (OA/A) molar ratio and clinical outcomes in Leptospirosis. Through a prospective observational cohort study employing high-performance liquid chromatography (HPLC) we sequentially determined serum concentrations of nonesterified fatty acids (NEFA) and albumin in twenty-eight patients with severe leptospirosis since their hospital admission. Twenty patients recovered, and eight died. Data was distributed in two groups according to clinical outcomes. Oleic acid/albumin molar ratios (OA/A), initial samples, were higher than those in healthy donors. The ratio OA/A, however, persisted high in dying patients, whereas patients who survived had a reduction matching to healthy donors. Biochemical alterations suggest that cure is correlated to the reestablishment of the OA/A molar ratio, while fatal outcomes related to persisting OA/A imbalances. Analysis by receiver operating characteristic (ROC) showed the area under the curve of 0.864 and the cutoff value of 0.715 being associated with a high odds ratio. Lipid analysis from patients with leptospirosis had an acute high serum OA/A molar ratio, and sustained imbalance has a high odds ratio and strong correlation with mortality.

## 1 Introduction

Lipotoxicity refers to functional impairments associated with increased lipids levels, and their fatty acids (FA) induced metabolic alterations and intracellular signaling (Wende et al., 2012; Engin 2017). Lipid changes in infectious diseases have critical effects on various stages of host-pathogen interactions (van der Meer-Janssen et al., 2010). Lipidomics has reported lipid compositions of some pathogens and their effectiveness in microbial pathogenesis (van der Meer-Janssen et al., 2010). Lipids have ubiquitous functions for humans, making them an attractive target for microbes (Walpole et al., 2018). Cells from the innate immune system engulf microbes and destroy them (Walpole et al., 2018). However, some pathogens developed mechanisms to evade the immune system, allowing survival and multiplication (Hsiao et al., 1991). *Leptospira* presents peculiar characteristics in its cellular lipid composition, and in a sophisticated way, its glycolipoprotein (GLP) endotoxin fits this pathophysiological pattern (Goncalves-de-Albuquerque et al., 2012; Goncalves-de-Albuquerque et al., 2014a, b).

Leptospirosis, a worldwide zoonosis caused by the Leptospira genus' pathogenic spirochetes, presents multiple organ involvement and high lethality (Bharti et al., 2003). Patients with leptospirosis present metabolic disturbance with dyslipidemia with a rise in blood NEFA levels and OA/A and oleic + linoleic/albumin molar (OA + L/A) ratios imbalance, which correlates with the disease severity in such patients (Burth et al., 2005; Gazi et al., 2011). GLP cellular physiopathology is complex and involves immune system activation through Toll-like receptor-2 (TLR2) (Younes-Ibrahim et al., 1997; Lacroix-Lamande et al., 2012) and TLR4 (Werts et al., 2001; Goncalves-de-Albuquerque et al., 2015) activating intracellular pathways leading to an inflammatory microenvironment and organ damage. The finding that GLP increases the apparent affinity of Na/K-ATPase for sodium (Brandao et al., 1998, Park, Goodman et al.) suggested that it might interact with the sodium-binding sites of Na/K-ATPase, on the cytosolic surface of cell membranes, potentially by acting of their lipid component in the cell membrane. The lipid content (Oleic acid) acts through the Na/K-ATPase inhibition independently of Toll-like receptor activation (Goncalves-de-Albuquerque et al., 2013).

For the first time, the present study approaches the correlation between OA/A molar ratios and clinical outcomes of hospitalized patients with leptospirosis and Weil's syndrome.

## 2 Material and methods

### 2.1 Study design

We carried out a cross-sectional comparative study to explore acute lipotoxicity factors associated with Weil's disease outcomes among hospitalized patients with serologically confirmed leptospirosis from January 2008 to December 2011.

### 2.2 Patients and inclusion criteria

All patients had confirmed leptospirosis by macroscopic-agglutination test (Brandao et al., 1998). Duplicate serum samples were sequentially collected and kept frozen at -70 °C until needed for chromatography. We studied eighty-four samples from 28 patients with severe forms of leptospirosis requiring hospitalization. Serum samples from ten healthy were used as the control group. Samples were collected every 48 h during hospitalization, and the last collection was performed within 24 h prior to discharge or death. All patients received antibiotic therapy and careful crystalloid fluid replacement. The mean time (±SD) of hospitalization was 7 ± 3 days in group 1 and 4 ± 1.5 days in group 2.

The selected patients were admitted with suspected Weill syndrome in the ICU. Serological confirmation was made later. All patients had kidney impairment (mean serum creatinine of 4.6 ± 2.5 mg/dL) and ARDS (mild and severe) according to Berlin definition (Matthay et al., 2012) and had abnormal liver function assay. Table 1 shows the patient's clinical and laboratory.

**Table 1:** Clinical findings of patients.

	n	%
Male, n	19	68
Age (years)	39 ± 25	
Jaundice	28	100
Fever	28	100
Onset of the syntoms to admission (days)	7 ± 3	
Length of hospital stay (days)	6 ± 1,5	
Pulmonary involvement	28	100
Breathness	28	100
Haemoptysis	5	18
Acute respiratory distress syndrome	9	32
Kidney failure	28	100
Oliguria	10	35
Haemodialysis	10	35
Neurological involvement	0	0
Cardiovascular involvement	3	10
Hemorrhage of any kind	10	35
Cure	20	71
Death	8	29
Laboratory findings (Mean ± SD %)	Mean	±SD %
Hemoglobin	9,8	0,9
White blood count (X10 3/mm3)	14,2	5
Platelet count (X10 3/mm3)	48,5	61
Serum potassium mEq/L	3,5	0,7
Serum creatinine (mg%)	4,6	2,5
AST (IU/L)	130	86
ALT (IU/L)	70	28
Direct bilirubin (mg%)	12	8
Lactat dehydrogenase IU/L	845	277

### 2.3 Ethics statement

The research protocol (2430-CEP/HUPE) was accepted by the Hospital Universitário Pedro Ernesto Ethics Committee under the number 14017313.4.0000.5259, and it was done following the Declaration of Helsinki. Informed oral consent was obtained from patients. The use of oral consent was approved as it was thought to be appropriate for this observational study. Oral consent was witnessed and documented on a formulary. By resolution of the Brazilian National Board of Health (1996), the term of free and informed consent in research protocols involving children and adolescents requires informed consent by the legal guardian of the subject without suspension of the individual's right to information to the extent of their ability. All necessary parental consent for this study was obtained, and data were anonymously analyzed.

### 2.4 Serum albumin assays

Serum albumin was quantified, according to Doumas et al. (Doumas et al., 1971).

### 2.5 Serum oleic acid quantification

Oleic acid serum levels were determined by high-performance liquid chromatography (HPLC). Fatty acid extraction was done according to Puttman et al. (Puttmann et al., 1993, Goncalves de Albuquerque et al., 2012). Briefly, lipids were obtained from 100 μL serum samples. We added an internal standard the margaric acid and performed the extraction and derivatization. Obtained fatty acids were dissolved in acetonitrile and injected onto a reverse phase, free fatty acid HP 3.9 × 150 mm column (Waters Corporation). The UV detection was performed at 254 nm. Standard solutions of fatty acids derivatives were used to calibrate the system.

### 2.6 Statistical analysis

Results are expressed as the mean ± standard error (SEM). The analysis of variance for multiple comparison Tukey-Kramer tests (ANOVA) was used to determine statistical significance of oleic acid/albumin molar ratios compared to controls. A 95% Confidence Interval (CI) was considered statistically significant. We performed the Deming Regression for Estimating Systematic Bias (Martin 2000) of OA/A ratio in all blood samples from both groups of patients during hospitalization to investigate the kinetics of OA/A ratios over time. A ROC curve was produced for OA/A molar ratios in serum samples from group 1 patients at hospital discharge and group 2 patients before death. ROC curve evaluated the specificity and sensitivity of the OA/A molar ratio and its association with the outcome of leptospirosis (p < 0.05 was considered significant) at the time of outcome. The program used for statistical analysis and graph creation was Graph Pad Prism 2.1 and Graph Pad Instat 3 (Graph Pad Software Inc). Woolf's method (Agresti and Min 2002) was used to estimate the odds ratio as well as positive (PPV) and negative predictive (NPV) values. Haldane's principle (Haldane 1956) was utilized to adjust the number of samples.

## 3 Results

### 3.1 Study cohort

We studied eighty-four samples from 28 patients (19 men and 9 women between 13 and 64 years old). All patients presented the severe form of leptospirosis requiring hospitalization and were analyzed in two groups: group 1 consisted of 20 patients (11 men and 9 women between 13 and 64 years old) with recovery outcomes, and group 2 consisted of 8 men (between 42 and 59 years old) with fatal outcomes. Serum samples from ten healthy volunteers (5 men and 5 women between 20 and 55 years old) were used as the control group.

### 3.2 Oleic acid/albumin molar ratios among groups

The Tukey-Kramer test was used to compare molar ratios expressed as the mean ± SD for samples collected at the beginning of hospitalization and the last samples collected.

**Figure 1:**
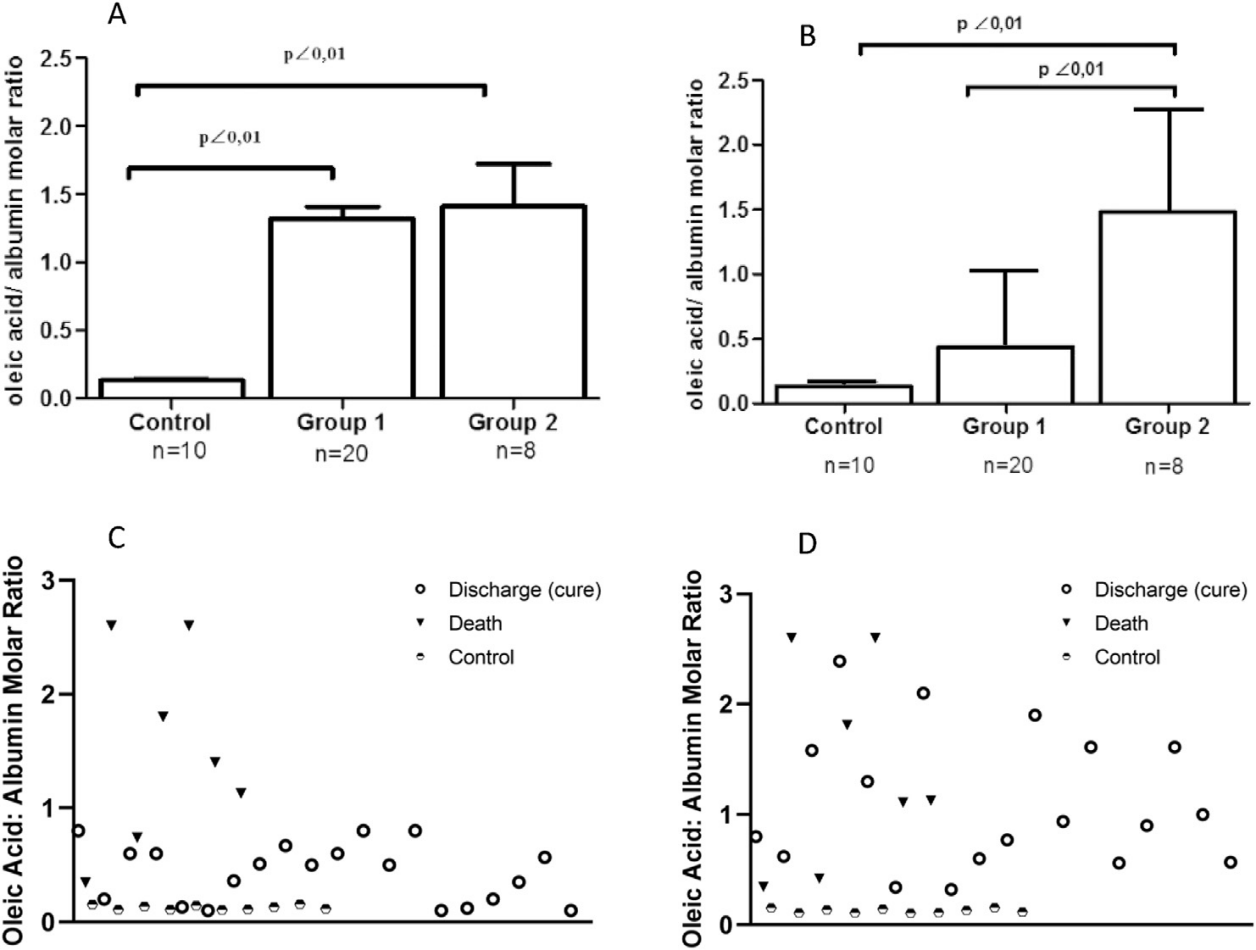
Oleic acid/albumin molar ratios by group: An analysis by the multiple Tukey-Kramer test. Oleic acid/albumin molar ratios at two time points: (A) at hospital admission: Group 1 (1.32 ± 0.39) and Group 2 (1. 41 ± 0.89) were significantly different from control (0,037 ± 0,058), with q = 6.412 (CI: -1.509 -0.45050) and q = 5.716 (CI: -1.718 -0.4216), respectively; p < 0.01. The groups 1 and 2 were not statistically significant q = 0.5150 (CI: -0.6619 0.4819). (B) Before hospital discharge: Group 1 (0.44 ± 0.58) and control (0.037 ± 0.058) were significantly different from Group 2 (1.48 ± 0.8) with q = 5.638 (-1.550 -0.3701) and q = 5.490 (-1.729 -0.3911), respectively; p < 0.01. When Group 1 and 2 were compared q = 0.643 (CI: -0.6461 to 0.4461); and p < 0.01. Individualized patients are showed in Figures 1C and 1D.

According to the clinical outcomes, biochemical patterns were different in the patient's group. The OA/A molar ratios were similarly high in both patient groups at hospital admission (P < 0.01) compared to the control group (Figure 1A). Molar ratios in group 2 were higher than group 1 at hospital discharge (P < 0.01). Conversely, in group 1, the OA/A decreased over time and was not significantly different from hospital discharge controls (Figure 1B). We also include individualized patient representation in Figures 1C and 1D.

### 3.3 Serum albumin analysis

**Figure 2:**
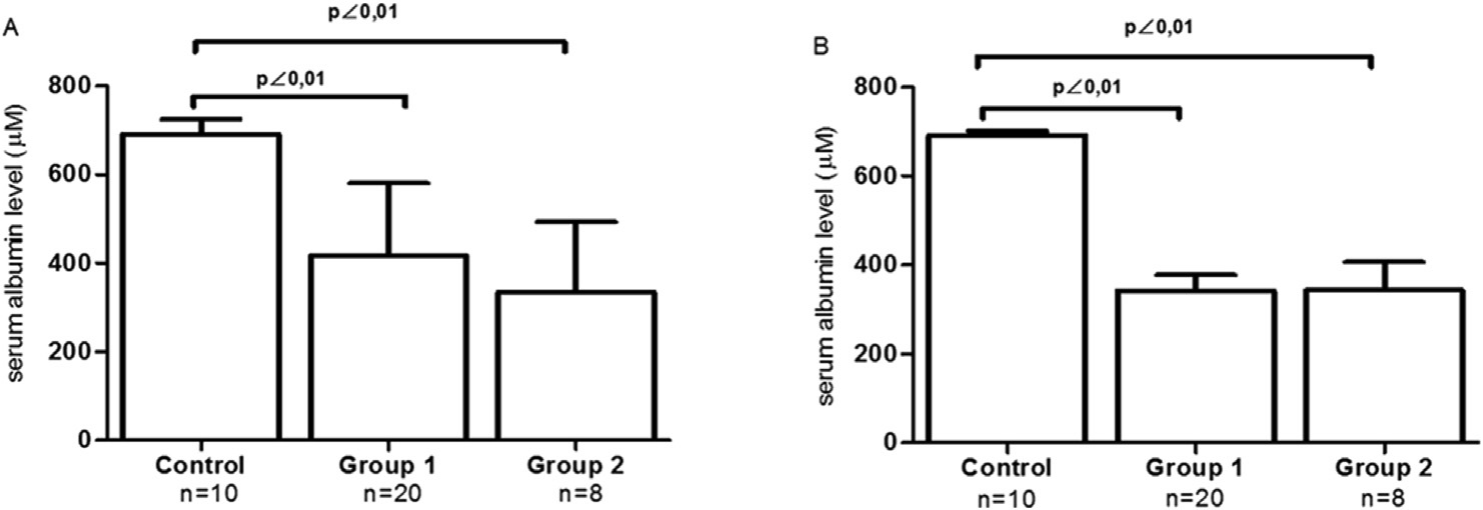
Serum albumin levels by the Multiple Tukey-Kramer test. (A) At hospital admission: Groups 1 (416 ± 164 μM) and 2 (334 ± 159 μM) both were significantly different from controls (691 ± 34 μM), p < 0.001 with q = 7.165 (CI: -407. 95 to -142.05) and 7.595 (CI: -357 to -519), respectively. When comparing groups 1 and 2, q = 1.978 (CI: -61.604 to -225.60); p > 0.05. (B) At the time of hospital discharge or prior to death: Group 1 (342 ± 156 μM) and Group 2 (343 ± 179 μM), presented p < 0.001 compared to controls (691 ± 34 μM) with q = 9.030 (CI: -482.88 to -215.12) and q = 7.352 (CI: -511.97 to -184.03), respectively; when compared, groups 1 and 2 showed q = 0.02395 (CI:-145.61 to 143.61), respectively; p > 0.05.

There was no distinct pattern in serum albumin levels between leptospirosis groups during the studied period. Group 1 albumin levels were statistically similar to group 2, and both were significantly lower compared to controls during the entire hospitalization time (Figure 2).

### 3.4 Deming Regression of Oleic Acid/Albumin molar ratios of patients through hospitalization time, according to prognosis

**Figure 3:**
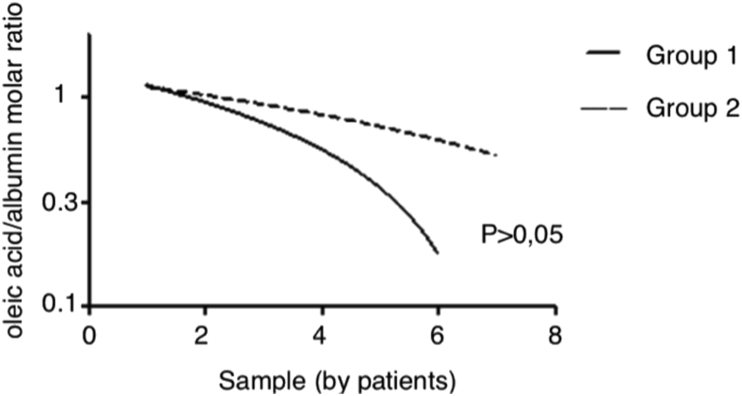
Deming Regression of Oleic Acid/Albumin molar ratios of patients through hospitalization time according to prognosis: Although OA/A of patients over time were different (the patients who died were hospitalized for a shorter time), we observed a statistically significant decrease in OA/A ratio over time in group 1, which did not occur in group 2. Group 1: F = 443057.62, (CI: -0.1511 to -0.003701), p < 0.05 and Group 2: F = 0.5782, (CI: -0.1209 to 0.02459).

Since OA/A of patients over time were different (patients who died were hospitalized for a shorter time), we performed the Deming Regression to minimize bias and to study the kinetics of OA/A in both groups during hospitalization. We observed a statistically significant decrease in OA/A in group 1, which did not occur in group 2 (Figure 3).

### 3.5 ROC curve of oleic acid/albumin molar ratios

**Figure 4:**
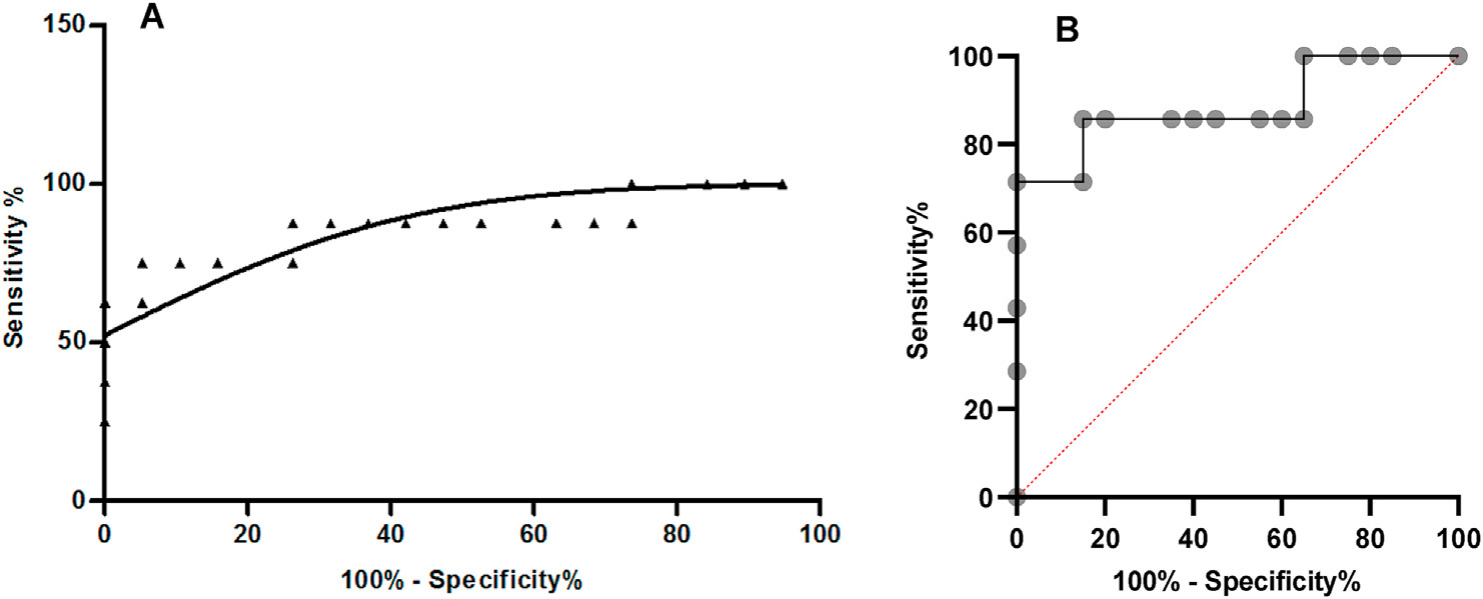
ROC curve for serum oleic acid/albumin molar ratios from leptospirosis patients according to the clinical outcome: AUC of 0.8684 (CI: 0.689 to 1.047); p = 0.0029 (A) and ROC curve with reference line (B).

Analysis by ROC curve (Figure 4A) showed an AUC of 0.8684 (0.689–1.047), p = 0.0029, and a cutoff value of 0.7150 for oleic acid/albumin molar ratios. These values are associated with high specificity (87.50%) and sensitivity (73.68%) for leptospirosis outcomes.

Using this cutoff and a 2 × 2 contingency table, the measured odds ratio (OD) was determined to be 25 (3.744–165), the chi-squared test (χ^2^) was 11.134 (p < 0.05), and the sensitivity and specificity were 0.825 and 0.850, respectively. PPV was 0.700 and NPV 0.9444. Therefore, a constant oleic acid/albumin ratio over 0.7150 may be considered a marker leading to leptospirosis mortality. We also added a ROC curve with a reference line (Figure 4B).

### 3.6 Linear regression comparing molar ration oleic acid/albumin

**Figure 5:**
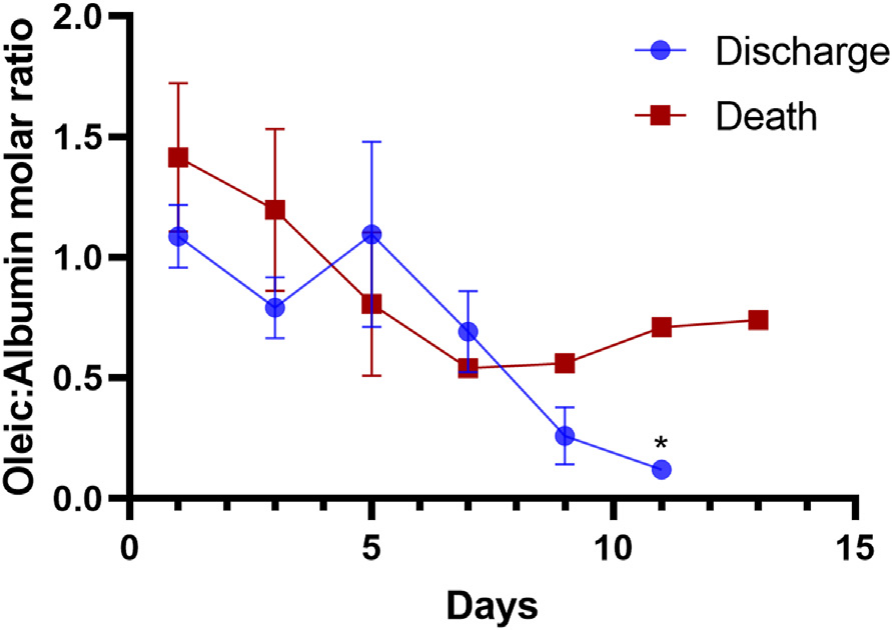
Linear Regression Oleic/Albumin molar ratio during hospitalization. The linear regression was made comparing oleic acid/albumin molar ration to two outcomes discharge (cure) or death. Molar ration from discharge patients present statistical difference with P value 0.0171.

We performed linear regression comparing the molar ratio oleic acid/albumin in two outcomes death or hospitalization discharge (cure). Patients who died had no change in AO/A over the days. The decrease in the ration OA/A was pronounced in surviving patients who healed (Figure 5). These data support the key role of oleic acid on disease outcome.

## 4 Discussion

Human Leptospirosis presents a wide variety of clinical manifestations, from a febrile syndrome to a fatal course. Some basic questions remain unclear, such as whether the clinical course depends on the virulence of the pathogen only or if it also depends on host immunity modulating biochemical repercussions. In leptospirosis, a broad spectrum of biological events seems to contribute to the disease pleomorphism. The LPS endotoxin does not have the same biological relevance seen in other Gram-negative bacteria and may be related to macrophage activation through TLR2 binding (Werts et al., 2001). The GLP endotoxin's specific action inhibiting Na/K-ATPase was demonstrated and potentially explains most acute cytotoxicity observed in different tissues during this disorder (Burth et al., 1997; Younes-Ibrahim et al., 1997, Lacroix-Lamande et al., 2012). The inflammasome seems to play a critical role in the host defense, and its activation ends up in the secretion of pro-inflammatory cytokines (Bauernfeind et al., 2011; Davis et al., 2011; van de Veerdonk et al., 2011). The inhibition of Na/K-ATPase, which occurs in leptospirosis, also triggers inflammasome activation (Lacroix-Lamande et al., 2012).

Lipids from host cells as well as from pathogens are critical to pathogens evade from the immune system. Pathogens evolved sophisticated mechanisms to exploit the lipid host metabolism to survive and replicate (Walpole et al., 2018). Pathogens use lipids as a nutrient source and alter host cell physiology to proliferate (van der Meer-Janssen et al., 2010).

The *Leptospira* have a peculiar lipid composition, and the NEFA constitutes part of the GLP glycolipoprotein endotoxin. Considering Na/K-ATPase as a molecular target for both GLP endotoxin and non-esterified unsaturated fatty acid (NEUFA) (Goncalves-de-Albuquerque et al., 2012, Lacroix-Lamande et al., 2012; Goncalves-de-Albuquerque et al., 2014a, b), synergistic inhibition of enzyme activity is a pathophysiological effect that could be predicted in severe leptospirosis. Data from our group using animal model strengthen this idea because of both GLP and oleic acid-induced lung injury in a similar fashion to the human form of ARDS (Goncalves-de-Albuquerque et al., 2012; Goncalves-de-Albuquerque et al., 2013; Goncalves-de-Albuquerque et al., 2014a, b; Goncalves-de-Albuquerque et al., 2015).

In 1971, Ahmed (Ahmed and Thomas 1971) first described the effect of *in vitro* NEUFA on Na/K-ATPase. Subsequently, this enzyme was shown to have an additional site where lipid second messengers could interact directly, changing cellular functions (Oishi et al., 1990; Hostmark 1995).

Increased serum NEFA is observed in various acute clinical conditions such as trauma, sepsis, pancreatitis, embolism, and pre-eclampsia (Bursten et al., 1996; Sztefko and Panek 2001; Turgut et al., 2015; Chung et al., 2017; Posti et al., 2017). Leptospirosis patients with pulmonary and renal complications are linked to a high mortality rate, and patients without acute kidney injury are most likely to survive (Daher Ede et al., 2010). Bursten et al. (Bursten et al., 1996) found that serum from seriously ill patients with sepsis, severe trauma, or ARDS had a disproportionate increase in C18 unsaturated free fatty acid (OA) concentrations, and at-risk patients who were destined to develop ARDS had higher levels than similar at-risk patients who did not develop ARDS (Herath et al., 2019). Experimentally, oleic acid intravenous administration induces ARDS in animal models (Goncalves-de-Albuquerque et al., 2012; Goncalves-de-Albuquerque et al., 2013; Goncalves-de-Albuquerque et al., 2015).

In previous work, we investigated the role of an acute increase in serum-free fatty acids levels observed in patients with Weil's disease, which can be represented mainly through OA/A and (OA + Linoleic/A) molar ratios and the serum albumin capacity to prevent *in vitro* NEUFA cytotoxic effects (Burth et al., 2005). We did not observe a statistical difference in linoleic/A molar ratios (data not shown). Leptospirosis patients present alteration in serum lipids and lipoproteins (Estavoyer et al., 1985), with increased triglycerides and very-low-density levels lipoprotein and a decrease in levels of high-density lipoprotein (Gazi et al., 2011). Here, we analyzed the correlation of OA/A molar ratios and the clinical outcomes of hospitalized patients and defined by OA/A ratio the cutoff of this relationship, the point at which there would be a higher likelihood of death as defined by the odds ratio. There was a decrease in OA/A ratios over time in a survival group, but not in patients who died.

When mortality vs. survival was compared, the patients who died were hospitalized for a shorter time. All patients who died were male over 40 years old and it is in line with previous publications linking fatal outcome to gender and age (Felzemburgh et al., 2014; Costa et al., 2015). To make proper corrections, we have performed the Deming Regression for Estimating Systematic Bias of OA/A in all blood samples from both groups of patients over time.

Serum albumin levels alone did not correlate with clinical outcome in this study. Albumin levels dropped quickly in critical cases, but slowly returned to normal levels in cured patients. A limitation about that is the relatively short hospitalization time (Wooley and Hunter 1970; Lefevre et al., 1988; Doweiko and Nompleggi 1991; Quinlan et al., 2004; Quinlan et al., 2005; Yu et al., 2017).

Each albumin molecule can bind up to seven free fatty acid molecules. Some deleterious effects have been described in situations where high NEUFA/albumin molar ratios could result from a low saturation capacity of the albumin binding sites (Hostmark 1995; Younes-Ibrahim et al., 1997; Goncalves-de-Albuquerque et al., 2012). In this regard, we developed a test to measure the fatty acid-albumin saturation to determine indirectly NEFA toxicity (Goncalves-de-Albuquerque et al., 2019). That test has a potential application to critically ill patients (Goncalves-de-Albuquerque et al., 2019). Even though there were no differences in albumin serum between patient's groups, the levels of NEFA were different, and it is the key point, because higher levels of NEFA may increase toxicity with a worse prognosis. During leptospirosis, patients have higher serum free fatty acid levels, decreased serum albumin, and elevated serum total bilirubin resulting from hepatic abnormalities. It has already been reported that bilirubin competes with NEFA for albumin binding sites (Martin and Lewis 2004), so it may also contribute to a lower albumin NEFA retaining capacity.

Despite the limited number of patients in these clinical observations, our results show a robust correlation. We showed that the OA/A molar ratio reestablishment to near control values was an excellent parameter indicating leptospirosis patient recovery (Burth et al., 2005). Nonetheless, the persistence of OA/A molar ratios above the 0.7150 cutoffs seems correlated to patient death. These findings suggest that OA/A molar ratio can be a potential NEUFA lipotoxicity biomarker. Therefore, we hypothesized that the plasma level of oleic acid and its relationship with albumin should be a biomarker of this dynamic pathophysiological mechanism involving lipid metabolism, justifying its correlation with the outcomes.

Albumin showed beneficial effects in reducing the inflammatory response and promoting decreased vascular permeability (Zhang and Frei 2002; Martin and Lewis 2004). Most likely, due to its antioxidant properties, albumin restored respiratory dysfunction in sepsis (Yu et al., 2017). Furthermore, albumin administration increased hemodynamic stability and oxygenation in patients with lung injury (Zhang and Frei 2002; Powers et al., 2003; American Thoracic 2004; Martin and Lewis 2004). Albumin's clinical and physiological benefits are not fully understood and appear to be directly related to its properties. Nevertheless, the correlation of its serum levels with the renal prognosis in critical patients seems to be already established (Yu et al., 2017).

This study's observations contribute to open new therapeutic perspectives for the potential parenteral albumin use to reduce circulating OA/A ratio and minimize the deleterious pathophysiological effects of systemic free oleic acid.

## 5 Conclusion

Lipidome from patients with leptospirosis revealed that the persistence of high serum OA/A molar ratios levels in infected patients showed both a strong odds ratio and a strong correlation with mortality. Conversely, patients with a lower OA/A ratio survive, and it should be considered a new potential prognostic biomarker factor for leptospirosis outcomes. Further studies are necessary to confirm our preliminary study.

## Declarations

### Author contribution statement

Mauro Velho Castro Faria and Mauricio Younes-Ibrahim: Conceived and designed the experiments; Analyzed and interpreted the data; Wrote the paper.

Caroline Azevedo Martins and Cassiano Felippe Gonçalves-de-Albuquerque: Performed the experiments; Analyzed and interpreted the data; Wrote the paper.

Maria Conceição Bastos Santos: Performed the experiments; Analyzed and interpreted the data.

Patricia Burth and Hugo Castro-Faria-Neto: Performed the experiments; Analyzed and interpreted the data; Contributed reagents, materials, analysis tools or data.

### Funding statement

This work was supported by Fundação Carlos Chagas Filho de Amparo à Pesquisa do Estado do Rio de Janeiro (FAPERJ) Grants (E-26/010.000983/2019, E-26/203.290/2017, and E-26/2010.592/2019), 10.13039/501100003593Conselho Nacional de Desenvolvimento Científico e Tecnológico (CNPq), Programa Estratégico de Apoio à Pesquisa em Saúde (PAPES-FIOCRUZ), and 10.13039/501100002322Coordenação de Aperfeiçoamento de Pessoal de Nível Superior (CAPES) - Grant 001, the European Community’s Seventh Framework Programme (FP7-2007-2013) under grant agreement HEALTH-F4-2011-282095 (TARKINAID), Programa de Produtividade Científica da Universidade Estácio de Sá, Programa de Pós Graduação em Biologia Molecular Celular (UNIRIO), Programa de Pós Graduação em Ciências e Biotecnologia (UFF), the Programa de Pós-Graduação em Fisiopatologia Clínica e Experimental – FISCLINEX, Faculdade de Ciências Médicas da Universidade do Estado do Rio de Janeiro (UERJ), and Pontifícia Universidade Católica do Rio de Janeiro.

### Data availability statement

Data included in article/supplementary material/referenced in article.

### Declaration of interests statement

The authors declare no conflict of interest.

### Additional information

No additional information is available for this paper.
